# Role of mitochondrial processing peptidase and AAA proteases in processing of the yeast acetohydroxyacid synthase precursor

**DOI:** 10.1002/2211-5463.12088

**Published:** 2016-06-10

**Authors:** Suvarna Dasari, Ralf Kölling

**Affiliations:** ^1^ Institut für Lebensmittelwissenschaft und BiotechnologieFg. Hefegenetik und Gärungstechnologie (150f)Universität HohenheimStuttgartGermany

**Keywords:** acetohydroxyacid synthase, i‐AAA, m‐AAA, mitochondrial import, mitochondrial processing peptidase, presequence processing

## Abstract

We studied presequence processing of the mitochondrial‐matrix targeted acetohydroxyacid synthase (Ilv2). C‐terminal 3HA‐tagging altered the cleavage pattern from a single step to sequential two‐step cleavage, giving rise to two Ilv2‐3HA forms (A and B). Both cleavage events were dependent on the mitochondrial processing peptidase (MPP). We present evidence for the involvement of three AAA ATPases, m‐ and i‐AAA proteases, and Mcx1, in Ilv2‐3HA processing. Both, precursor to A‐form and A‐form to B‐form cleavage were strongly affected in a *∆yme1* mutant. These defects could be suppressed by overexpression of MPP, suggesting that MPP activity is limiting in the *∆yme1* mutant. Our data suggest that for some substrates AAA ATPases could play an active role in the translocation of matrix‐targeted proteins.

**Abbreviations**
AAA: ATPases associated with diverse cellular activities
CHX: cyloheximide
HA: hemagglutinin tag
i‐AAA: AAA protease with active site exposed to the intermembrane space
IMS: intermembrane space
m‐AAA: AAA protease with active site exposed to the matrix
MIP: mitochondrial intermediate peptidase
MPP: mitochondrial processing peptidase
MTS: mitochondrial targeting sequence
PP: precursor
TIM: translocase of the inner membrane
TOM: translocase of the outer membrane

Acetohydroxyacid synthase (Ilv2) catalyzes the first step in the biosynthesis of branched‐chain amino acids 1, which mainly takes place in the mitochondrial matrix 2. The nuclear encoded Ilv2 is synthesized in the cytoplasm with an N‐terminal mitochondrial targeting sequence (MTS) directing import into the mitochondrial matrix 3. Matrix‐targeted proteins are recognized by receptors on the surface of mitochondria and are imported through multi‐subunit translocases of the outer membrane (TOM) and inner membrane (TIM) into the mitochondrial matrix 4. The presequences of matrix‐targeted proteins are cleaved off by mitochondrial processing peptidase (MPP), a conserved hetero‐dimeric metallopeptidase, which in yeast consists of the subunits Mas1 and Mas2 5. Preprotein cleavage does not appear to be essential for translocation into the matrix, since import continues in the absence of processing 6. But, processing seems to be important for the activity of at least some proteins, since yeast cells are not viable in the absence of MPP 7. Also, nonprocessed precursor proteins are less stable, which could explain why MPP activity is essential 8. In some cases, additional sequences are removed after MPP cleavage. The mitochondrial intermediate peptidase (MIP) removes an octapeptide from the preproteins after MPP processing 9, the protease Icp55 removes one amino acid 10. These additional cleavage events could play a role in regulating the stability of the proteins.

A certain fraction of Ilv2 seems to be localized to the cytoplasm 11. Since the N‐terminal extension of Ilv2 is unusually long compared with other matrix‐targeted proteins (90 aa) 10, it could play a role in regulating the distribution between cytoplasm and mitochondrial matrix. Therefore, the N‐terminal processing of the Ilv2 preprotein was examined more closely. N‐terminal processing of Ilv2 was altered by C‐terminal tagging. In contrast to native Ilv2, which was cleaved only once, two‐step sequential cleavage was observed with C‐terminally tagged Ilv2. The cleavage events were dependent on MPP and the m‐ and i‐AAA proteases. Our results further suggest that MPP activity is limiting in the i‐AAA protease mutant *∆yme1*.

## Materials and methods

### Yeast strains, plasmids, and media

The yeast strains used are listed in Table 1. The yeast strains are derived from the CEN.PK and BY4741 backgrounds. Insertions and deletions were introduced into the yeast genome by one‐step gene replacement with PCR‐generated cassettes 12. The insertions and deletions were verified by PCR. For overexpression of *MAS1* and *MAS2*, the two genes were amplified by PCR from yeast chromosomal DNA (*MAS1*: pos. −630 to 1460; *MAS2*: pos. −630 to 1519) and cloned in tandem into the vector YEplac195 13 to give plasmid pRK1109. All cloned genes were sequenced to ensure that no PCR mutations occurred. For standard experiments, yeast cells were grown in YPD medium (1% yeast extract, 2% bacto peptone, 2% glucose) or in SD/CAS medium (0.67% yeast nitrogen base w/o amino acids, 1% casein hydrolysate, 2% glucose).

**Table 1:** Yeast strains

Strain	Genotype	Source
BY4741	*MATa his3‐∆1 leu2∆ met15∆ ura3∆*	EUROSCARF
CEN.PK K61	*MATa leu2‐3,112 trp1‐289*	M. Ramezani‐Rad, Düsseldorf
MY111‐2	*MATa ade2 his3 ura3 mas1 (ts)*	E. Pratje, Hamburg
RKY2199	*MATa leu2‐3,112 trp1‐289 ILV2‐3HA::kan*	This study
RKY2326	*MATa leu2‐3,112 trp1‐289 ILV2‐3HA::kan ∆yta10::TRP1*	This study
RKY2329	*MATa leu2‐3,112 trp1‐289 ILV2‐3HA::kan ∆yme1::TRP1*	This study
RKY2330	*MATa leu2‐3,112 trp1‐289 ILV2‐3HA::kan ∆mcx1::TRP1*	This study
RKY2342	*MATa leu2‐3,112 trp1‐289 ∆ilv2::kan*	This study
RKY2374	*MATa ade2 his3 ura3 mas1 (ts) ILV2‐3HA::kan*	This study
Y00148	*MATa his3‐∆1 leu2∆ met15∆ ura3∆ ∆yta10::kanMX4*	EUROSCARF
Y03367	*MATa his3‐∆1 leu2∆ met15∆ ura3∆ ∆mcx1::kanMX4*	EUROSCARF
Y07144	*MATa his3‐∆1 leu2∆ met15∆ ura3∆ ∆yme1::kanMX4*	EUROSCARF

### Cycloheximide (CHX) chase

Yeast cultures were grown overnight to exponential phase (OD_600_ ≤ 1.0) in YPD medium. CHX was added to a final concentration of 0.1 mg·mL^−1^ to the culture. A constant amount of culture was harvested at 20 min intervals and cell extracts were prepared by glass‐beading in lysis buffer (50 mm HEPES, pH 7.4; 0.3 m sorbitol; 10 mm NaN_3_; with protease inhibitors) and analyzed by western blotting with anti‐Ilv2 or anti‐HA antibodies. Bound antibodies were detected with the Lumi‐Light Kit (Roche, Mannheim, Germany) and luminescence signals were recorded with the LAS‐3000 imaging system (FUJI, Kleve, Germany). Digital images of immunoblots were analyzed using ImageJ 1.47v (http://rsb.info.nih.gov/ij/). All experiments were performed at least twice, with the same result. A representative experiment is shown in each case.

### Kinetic model

We hypothesized that the Ilv2 preprotein cleavages are sequential. Our system is therefore an example of three consecutive, irreversible, homogeneous first‐order reactions in a closed system:

$$P\overset{k_{pa}}{\to }A\overset{k_{ab}}{\to }B\overset{k_{bx}}{\to }X.$$

(*P* is the precursor, *A* is the Ilv2 A‐form and *B* is the Ilv2 B‐form, *X* degradation products).

The rate equation connects the reaction rate with concentrations and constant parameters as follows:

$$P(t)=e^{-k_{pa}t}P_{0},$$

$$A(t)=\left(\frac{k_{pa}e^{-k_{ab}t}}{k_{pa}-k_{ab}}-\frac{k_{pa}e^{-k_{pa}t}}{k_{pa}-k_{ab}}\right)P_{0}+e^{-k_{ab}t}A_{0},$$

$$\begin{array}{cc} & B(t)=\left(\frac{k_{ab}k_{pa}e^{-k_{pa}t}}{k_{{pa}^{2}}+\left(-k_{bx}-k_{ab}\right)k_{pa}+k_{ab}k_{bx}}\right)P_{0}\\ & \;\;-\left(\frac{k_{ab}k_{pa}e^{-k_{bx}t}}{\left(k_{bx}-k_{ab}\right)k_{pa}-k_{bx}^{2}+k_{ab}k_{bx}}\right)P_{0}\\ & \;\;+\left(\frac{k_{ab}k_{pa}e^{-k_{ab}t}}{(k_{bx}-k_{ab})\left(k_{pa}-k_{ab}\right)}\right)P_{0}\\ & \;\;+e^{-k_{bx}t}B_{0}+\left(\frac{k_{ab}e^{-k_{ab}t}}{k_{bx}-k_{ab}}-\frac{k_{ab}e^{-k_{bx}t}}{k_{bx}-k_{ab}}\right)A_{0} \end{array}.$$

However, we observed, that *k*_*pa*_ ≫ *k*_*ab*_ for most of the measured samples. Hence, the precursor can be neglected as it vanishes very quickly. The solution simplifies to:

$$A(t)=e^{-k_{ab}t}A_{0},$$

$$B(t)=\left(\frac{k_{ab}e^{-k_{ab}t}}{k_{bx}-k_{ab}}-\frac{k_{ab}e^{-k_{bx}t}}{k_{bx}-k_{ab}}\right)A_{0}+e^{-k_{bx}t}B_{0}.$$

The matrix exponential was performed with GNU Maxima (http://maxima.sourceforge.net) and the exchange rate constants were estimated by utilizing this solution and applying a least square approach to the measured data as implemented in Octave‐Forge (http://octave.sourceforge.net).

## Results

### Ilv2 presequence processing is altered by C‐terminal tagging

**Figure 1:**
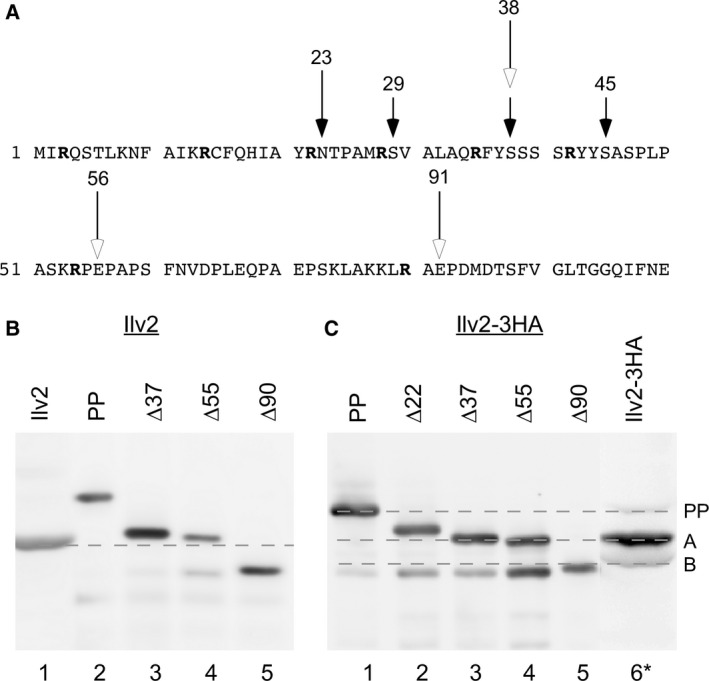
Processing of Ilv2 and Ilv2‐3HA. (A) Ilv2 presequence. Cleavage sites predicted by TargetP (37↓38) and MitoProt (55↓56), start of homology with *E. coli* acetohydroxyacid synthase (↓91) (open arrows), N‐termini detected by 10 (closed arrows). Arginine residues are highlighted (B) Mobility of Ilv2 from a CEN.PK K61 cell extract on an SDS/PAGE gel (1) compared to N‐terminally truncated marker proteins expressed in *E. coli* from the plasmids pRK1283 (2), pRK1284 (3), pRK1285 (4), and pRK1286 (5). Bands detected by western blotting with anti‐Ilv2 antibodies. (C) Same as in (B) but with C‐terminal 3HA‐tagged variants. (1) pRK1675, (2) pRK1676, (3) pRK1677 (4) pRK1678, (5) pRK1679, (6) RKY2199 extracts (Ilv2‐3HA, *signal enhanced). Bands detected by western blotting with anti‐HA antibodies. The sizes of the N‐terminal deletions are indicated, PP = precursor.

Potential presequence cleavage sites are predicted between aa 37 and 38 by the program TargetP 14, between aa 55 and 56 by MitoProt 15, and a study involving the whole yeast mitochondrial proteome places the main cleavage site between aa 22 and 23 10 (Fig. 1A). To see which of these cleavage sites are used in our yeast strain, we compared the mobility of Ilv2 from yeast cell extracts on SDS/PAGE gels with the mobility of N‐terminally truncated Ilv2 marker proteins expressed in *Escherichia coli*. One main Ilv2 band was detected that migrated slightly faster than the Ilv2∆55 variant (Fig. 1B), which places the mature N‐terminus a few amino acids downstream from the predicted 55↓56 site. In addition, a faint band corresponding to the Ilv2 precursor was detected.

We also performed experiments with a C‐terminally 3HA‐tagged Ilv2 variant. Strains expressing tagged Ilv2 grew like wild‐type on media lacking isoleucine, leucine, and valine proving that the protein is functional. Unexpectedly, a different banding pattern was observed with the tagged protein. In contrast to native Ilv2, two bands (designated A and B) and a faint precursor band were observed (Fig. 1C). The mobility of the A‐band was close to the mobility of the ∆37 and ∆55 forms. The resolution of our gel system is not good enough to clearly assign the A‐band to either one of these two forms. But, the A‐form clearly runs faster than the ∆22 variant. Thus, the mobility of the A‐form is compatible with cleavage between aa 37 and 38, and between aa 55 and 56. The B‐form matches most closely the ∆90 deletion.

We then investigated the contribution of MPP and AAA proteins to llv2 cleavage. Two hexameric AAA proteases are present in the inner mitochondrial membrane, one with the catalytic domain pointing toward the matrix (m‐AAA) and one with the catalytic domain pointing toward the intermembrane space (i‐AAA) 16. Another AAA‐protein (Mcx1) is the yeast homologue of bacterial ClpX that assists the ClpP protease in substrate binding and degradation 17. Since MPP activity is essential in yeast, a conditional temperature sensitive *mas1* mutant 7 was utilized to assess the role of MPP in Ilv2 processing.

**Figure 2:**
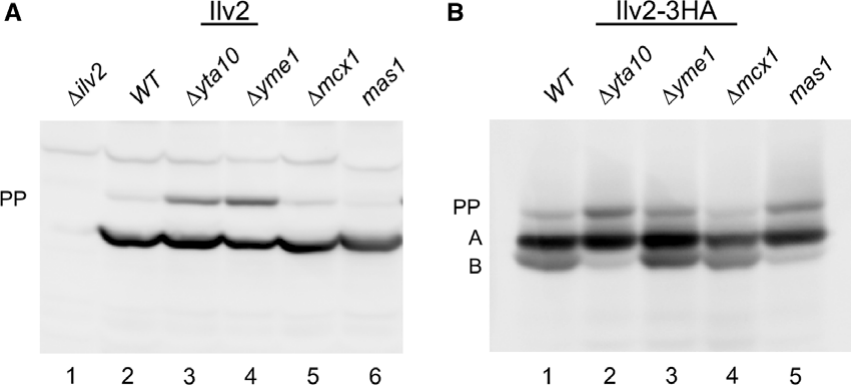
Effect of MPP‐ and AAA‐protein mutants on Ilv2 processing. Ilv2 and Ilv2‐3HA extracted from different yeast strains detected by western blotting with anti‐Ilv2 or anti‐HA antibodies. (A) Native Ilv2: (1) RKY2342 (*∆ilv2*), (2) BY4741 (*WT*), (3) Y00148 (*∆yta10*), (4) Y07144 (*∆yme1*), (5) Y03367 (*∆mcx1*), (6) MY111‐2 (*mas1*). (B) Ilv2‐3HA: (1) RKY2199 (*ILV2‐3HA*), (2) RKY2326 (*ILV2‐3HA ∆yta10*), (3) RKY2329 (*ILV2‐3HA ∆yme1*), (4) RKY2330 (*ILV2‐3HA ∆mcx1*), (5) RKY2374 (*ILV2‐3HA mas1*). PP = precursor, A‐ and B‐form see text.

With native Ilv2, deletion of m‐AAA (*∆yta10*) and i‐AAA (*∆yme1*) subunits led to an accumulation of the Ilv2 precursor band (Fig. 2A) pointing to a role of these proteins in Ilv2 translocation or processing. Deletion of *MCX1* and the *mas1* mutant at permissive temperature (25 °C) had no discernible effect on the Ilv2 banding pattern. Deletion of *YTA10* and *YME1* also affected processing of Ilv2‐3HA (Fig. 2B). Similar to native Ilv2, an accumulation of the precursor band was observed. An additional phenotype was observed for the *YTA10* deletion and the *mas1* mutant (grown at permissive temperature). In both cases, the B‐band was barely detectable indicating that the A to B conversion is compromised in these mutants. The A to B ratio in *∆yme1* and *∆mcx1* resembled wild‐type.

### Kinetics of Ilv2 processing

**Figure 3:**
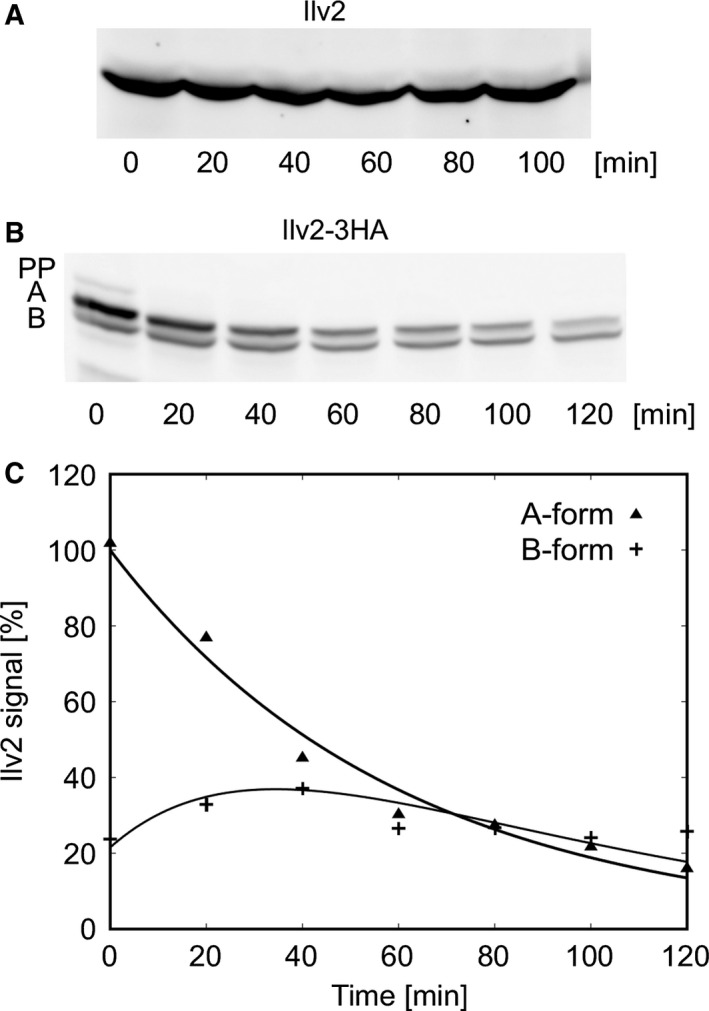
Two‐step processing of the Ilv2‐3HA preprotein. The half‐lives of the Ilv2 forms were determined by CHX‐chase. Samples were taken at the time intervals indicated. (A) CEN.PK K61 (*ILV2*), (B) RKY2199 (*ILV2‐3HA*), Ilv2 forms: preprotein (PP), A‐form (A), B‐form (B). (C) The signal intensities of the Ilv2‐3HA bands from (B) were quantified with the program ImageJ and plotted against the chase period. The signal intensities were normalized to the intensity of the A‐form at *t*_0_. The solid lines represent the prediction from the sequential kinetic model. The actual data points are: A‐form (filled triangles), B‐form (plus).

We wanted to know whether the two Ilv2‐3HA forms result from independent or sequential cleavage events. To this end, the turnover of the various Ilv2 forms was examined by cycloheximide (CHX)‐chase. The mature form of native Ilv2 proved to be fairly stable. No significant turnover was observed within 100 min (Fig. 3A). Ilv2‐3HA, however, behaved differently. At *t*_0_, the precursor band was barely visible and disappeared quickly during the chase period, the A‐form decreased steadily with time, while the amount of the B‐form stayed more or less constant (Fig. 3B). To determine the half‐lives of these two Ilv2 forms, we set up a kinetic model assuming two sequential cleavage events followed by degradation of the B‐form (PP→A→B→X) and first‐order kinetics for all reactions. The simulations showed a perfect match to the experimental data (Fig. 3C). We therefore conclude that the Ilv2‐3HA cleavages occur sequentially. The A‐form had a half‐life of 37 min and the B‐form had a half‐life of 24 min (Table 2).

**Table 2:** Turnover of Ilv2‐3HA forms

Strain	*k*_*ab*_ a	τ_A_ (min)^b^	*k*_*bx*_ a	τ_B_ (min)^b^
Wild‐type	0.0189	37	0.0287	24
*∆yta10*	0.00575	121	0.0437	16
*∆yme1*	0.00599	116	0.00979	71
*∆yme1+2μ‐MAS1,2*	0.00161	43	0.0231	30
*∆mcx1*	0.00503	138	0.00679	102

^a^ Rate constants for A to B conversion and B degradation, ^b^ half‐lives of A‐ and B‐forms.

**Figure 4:**
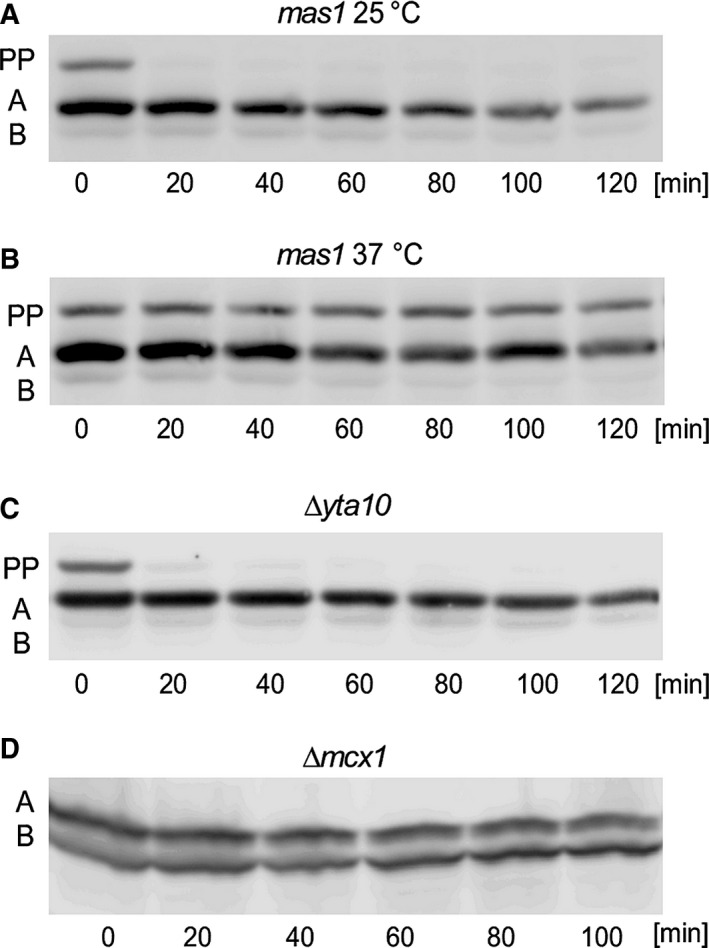
Stability of Ilv2 forms in different mutants. The half‐lives of the Ilv2 forms were determined by a CHX‐chase experiment. (A) RKY2374 (*mas1*, 25 °C), (B) RKY2374 (*mas1*, 37 °C), the cultures were pregrown at 25 °C and then shifted to 37 °C for 1 h before CHX addition, (C) RKY2326 (*∆yta10*), (D) RKY2330 (*∆mcx1*). Ilv2 forms: preprotein (PP), A‐form (A), B‐form (B). The proteins were detected by western blotting with anti‐HA antibodies.

Next, the turnover of the Ilv2‐3HA forms was examined in the *mas1* and in the AAA‐protein mutants by CHX‐chase (Fig. 4A). In the *mas1* mutant at permissive temperature (25 °C) at *t*_0_, a clear precursor band (PP), a strong A‐band signal, and only a very faint B‐band signal were observed as described above. The deviation from the wild‐type pattern suggests that MPP activity is already compromised at permissive temperature in the *mas1* mutant. After shifting to nonpermissive temperature (37 °C), the precursor band was completely stable (Fig. 4B). Since PP to A conversion is completely blocked and since the amount of B‐form is very low in the mutant, MPP seems to be involved in both PP→A and A→B cleavage events.

The *YTA10* deletion mutant showed a similar pattern as the *mas1* mutant at permissive temperature (Fig. 4C). The A‐form was strongly stabilized (τ = 121 min), while the turnover of the B‐form was not affected (τ = 16 min) (Table 2). An interesting pattern was observed for the *∆mcx1* mutant, here both forms were stabilized (τ = 138 min and τ = 102 min) (Fig. 4D). Since both forms are equally stabilized, the ratio of the two forms is similar to the wild‐type ratio, which explains why no clear difference to wild‐type in the banding pattern could be observed under steady state conditions (Fig. 2B).

### MPP activity is limiting in the *∆yme1* mutant

**Figure 5:**
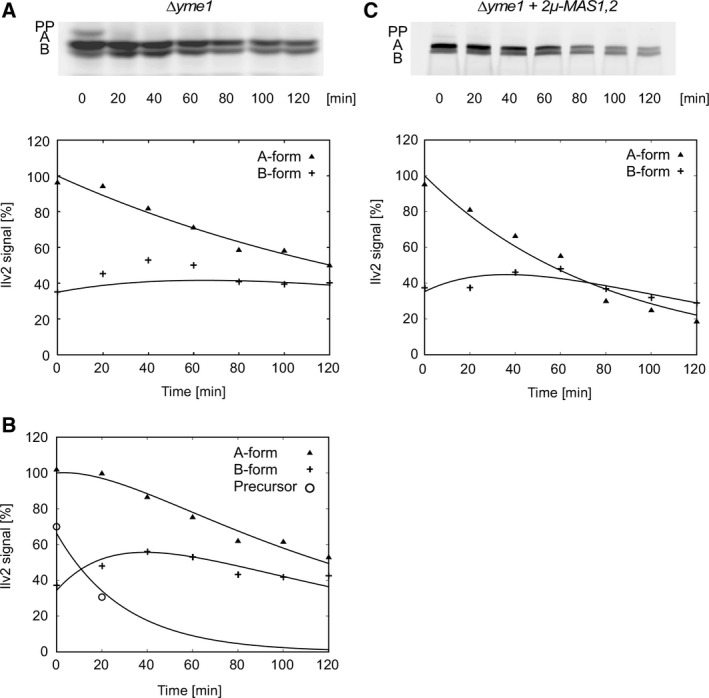
Effect of *∆yme1* on Ilv2‐3HA processing. The half‐lives of the Ilv2‐3HA forms were determined by CHX‐chase. (A) RKY2359 (*∆yme1*), detection of Ilv2‐3HA forms by western blotting with anti‐HA antibodies (upper panel), signal intensities plotted against the chase period (lower panel). The signal intensities were normalized to the intensity of the A‐form at *t*_0_. The solid lines represent the prediction from the sequential kinetic model. The actual data points are: A‐form (filled triangles), B‐form (plus). For the B‐form, the data points cannot be fitted to the sequential model. (B) Kinetic model incorporating direct PP to B cleavage. Precursor (open circle). (C) Overexpression of *MAS1* and *MAS2* suppresses the Ilv2‐3HA processing defect of *∆yme1*. RKY2359 (*∆yme1*) transformed with pRK1109 (*2μ‐MAS1,2*). Upper panel: CHX‐chase and western blot with anti‐HA antibodies, lower panel: signal intensities plotted against the chase period fitted to the sequential model.

All our kinetic data obtained so far were compatible with our sequential cleavage model. However, when we examined the kinetics of Ilv2 processing in the *∆yme1* mutant, a deviation from the sequential cleavage model was observed (Fig. 5A). At earlier time points during the CHX‐chase, there was ‘too much B‐form’. The experimental data could not be fitted to our sequential cleavage model (Fig. 5A, middle panel). The simplest interpretation for the occurrence of this extra amount of B‐form in the *∆yme1* mutant is that part of the Ilv2 precursor is directly converted to the B‐form. If we incorporate this assumption into our kinetic model, the data can be perfectly fitted (Fig. 5A, lower panel). From this model, the half‐lives of the Ilv2 forms were calculated. It turned out that both A to B conversion (τ = 116 min) and B degradation (τ = 71 min) are slowed down in *∆yme1*. This again explains why the ratio of A‐ and B‐forms is not so much different from the wild‐type. (Fig. 2B).

One possible reason for the direct PP to B cleavage could be that MPP activity is limiting in the *∆yme1* mutant. Thus, PP to A conversion may occur less efficiently increasing the likelihood of direct B cleavage. In line with this interpretation, we also observed accumulation of some precursor protein in *∆yme1*. To test this assumption, the MPP subunits, Mas1 and Mas2 were overexpressed from a multicopy plasmid in the *∆yme1* mutant. By this means, the altered kinetics of Ilv2 processing could be completely reverted to a wild‐type pattern (Fig. 5C, Table 2). This finding indeed suggests that limiting MPP activity is responsible for the altered kinetics of Ilv2 processing in *∆yme1*.

## Discussion

Here, we examined the processing of the Ilv2 presequence and uncovered a role for AAA‐proteins in translocation/processing of Ilv2.

Mature Ilv2 migrates slightly faster on SDS gels than a truncated variant missing the first 55 amino acids. This suggests that either cleavage occurs downstream from the 55↓56 site predicted by MitoProt or that initial cleavage by MPP is followed by an additional proteolytic event. Direct cleavage by MPP between aa 55 and 90 seems unlikely because an arginine residue is usually present at the −2 position from the cleavage site (‘R‐2 rule’) 5, and there is no arginine residue present in this region.

C‐terminal tagging alters the Ilv2 presequence cleavage pattern. In contrast to native Ilv2, two cleavage products (A and B) were observed for C‐terminally 3HA‐tagged Ilv2. The A‐band could not be unequivocally assigned to either one of the two predicted MPP cleavage sites (37↓38 and 55↓56), since the corresponding truncated Ilv2 forms migrate very close together on SDS gels. The mobility of the A‐band is therefore compatible with cleavage at both predicted MPP cleavage sites. The mobility of the B‐band is compatible with cleavage at another potential MPP cleavage site between aa 90 and 91 (arginine at position −2). The homology to *E. coli* acetohydroxyacid synthase starts at position 91.

Cleavage occurs in a sequential manner. MPP seems to be involved in both cleavage events, since PP to A conversion is completely blocked in *mas1* at nonpermissive temperature and the B‐band is barely detectable already at permissive temperature. Why tagging interferes with Ilv2 processing is not clear. We could offer the following interpretation: Tagging slows down Ilv2 translocation. B cleavage occurs only upon prolonged association of the precursor protein with the translocation channel. If translocation is fast, cleavage occurs at the 55↓56 site followed by an additional proteolytic event giving rise to the native Ilv2 form. The cleavage pattern would thus reflect the speed of translocation.

In contrast to native Ilv2, Ilv2‐3HA is an unstable protein. The higher turnover could be the result of the altered presequence cleavage. For the subunit IV of cytochrome c oxidase (Cox4), it has been shown that differential processing of the preprotein affects its half‐life 9. Cox4 is subjected to two‐step processing by MPP and MIP. If the second cleavage by MIP is prevented, the protein becomes unstable. Furthermore, evidence has been presented that the N‐terminal amino acid generated by cleavage of the preprotein may determine the half‐life of the cleaved protein 10. Differential cleavage of the presequence could therefore be a mechanism to regulate the turnover of key metabolic enzymes. The Ilv2‐3HA B‐form is strongly stabilized in the *∆mcx1* mutant. This suggests that the ClpX homologue Mcx1 assists in the degradation of Ilv2‐3HA.

Ilv2 presequence processing is affected by loss of m‐AAA protease function. For native Ilv2 as well as Ilv2‐3HA, we observed an accumulation of the precursor band. Also we found that A to B conversion is slowed down in case of Ilv2‐3HA, whereas degradation of the B‐band seemed to be unaffected. The simplest interpretation of these findings is that the Ilv2‐3HA presequence is a substrate for m‐AAA cleavage. Alternatively, m‐AAA could be involved in Ilv2‐3HA translocation, e.g. by keeping the Ilv2‐3HA precursor in an unfolded, translocation competent conformation.

Although m‐AAA is mainly known as a protease that degrades a large number on non‐native membrane proteins, it also appears to have specific regulatory functions. It is required for the proteolytic activation of the mitochondrial ribosomal protein MrpL32, a crucial step in ribosome assembly 18. It also plays a role in the two‐step processing of cytochrome c peroxidase (Ccp1), a protein that is targeted to the intermembrane space (IMS) 19.

We found that the i‐AAA protease is also involved in A to B conversion (and in B degradation), but the mechanism appears to be different. In the i‐AAA mutant *∆yme1*, the Ilv2‐3HA preprotein processing is no longer purely sequential. Instead, we also observed direct precursor to B cleavage. This altered kinetics and the stabilization of A‐ and B‐forms in the mutant could be completely suppressed by the overexpression of MPP. From this, we conclude that MPP activity is limiting in the *∆yme1* mutant.

An active role of Yme1 in translocation of mitochondrial proteins has already been suggested by a previous study 20. There, it has been shown that Yme1 is required for the targeting of mammalian polynucleotide phosphorylase (PNPase) to the IMS in yeast. In line with our observations, it was also noted that in *∆yme1*, PNPase was no longer processed by MPP (which occurred normally in a wild‐type strain). Basically, there are two possibilities, why *∆yme1* is suppressed by the overexpression of MPP. Either Yme1 is required for full activity of MPP, or MPP as well plays an active role in translocation, and overexpression of MPP compensates for a reduction in translocation efficiency due to loss of Yme1.

**Figure 6:**
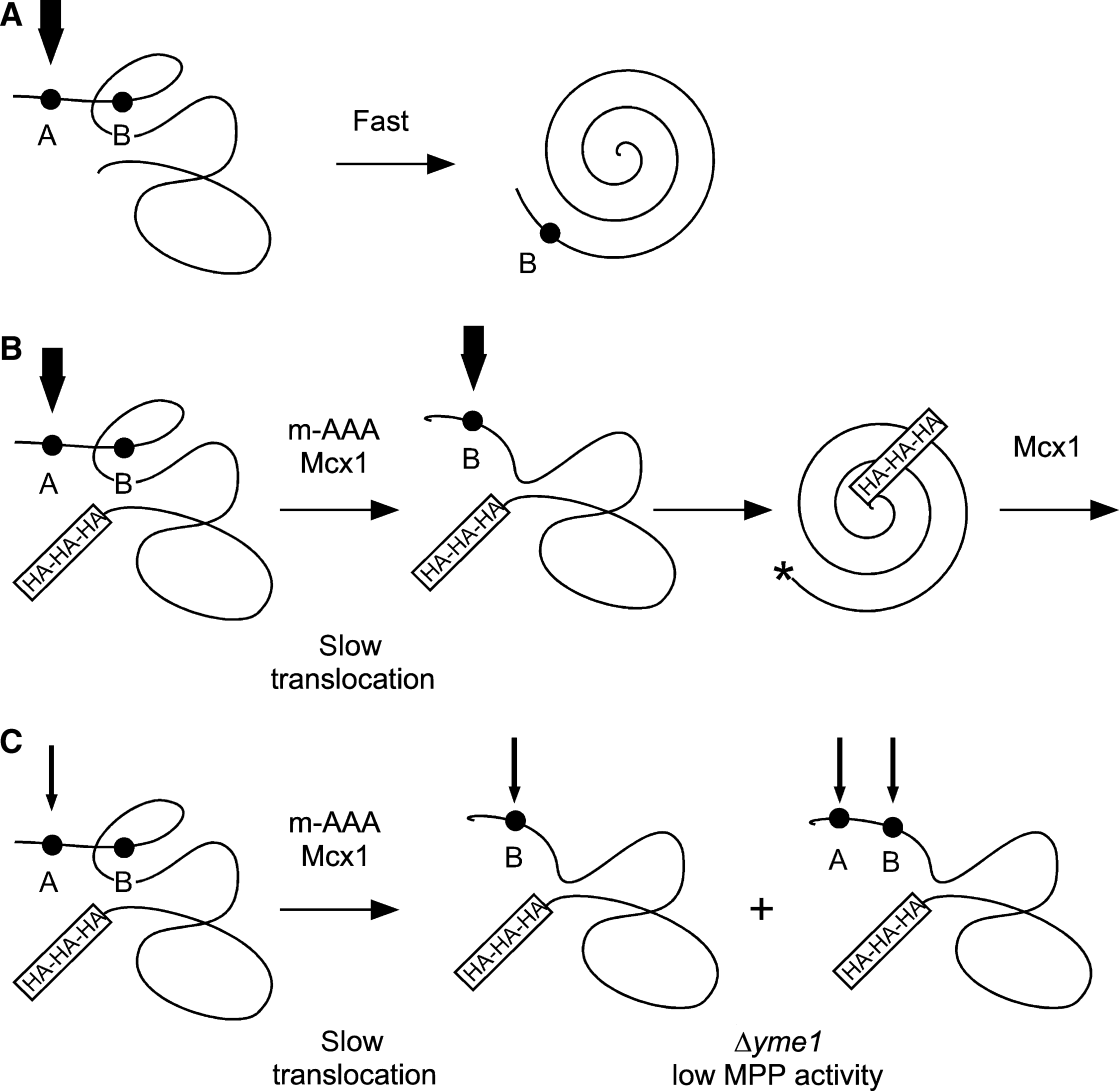
Model for Ilv2 processing. (A) Processing of wild‐type Ilv2. There are two sites for MPP cleavage, A and B. The precursor is cleaved at the A‐site as soon as it leaves the translocation channel. Rapid folding to the native conformation prevents further B‐site cleavage. (B) Processing of 3HA‐tagged Ilv2. C‐terminal tagging slows down translocation. At first, only the A‐site is exposed to the matrix and cleaved by MPP. Prolonged association with the translocation channel triggers association with m‐AAA and Mcx1. The AAA ATPases pull out the precursor from the translocation channel exposing the B‐site for MPP cleavage. The newly generated N‐terminus (asterisk) triggers Mcx1 dependent degradation. (C) Processing of 3HA in *∆yme1*. Similar situation as in (B), but with limiting MPP activity. Low MPP1 activity increases the likelihood of direct B‐site cleavage. Fat arrows: high MPP activity, thin arrows: low MPP activity.

Our data are summarized in the model shown in Fig. 6. It is not yet clear why the C‐terminal tag influences the N‐terminal processing, nor whether the effect is specific for a particular tag and/or for a single matrix protein. It remains to be seen whether the involvement in N‐terminal processing by the enzymes identified in the present work is widespread.

## Author contributions

SD performed the experiments, RK conceived and supervised the project and wrote the manuscript.
